# Low Cost Local Contact Opening by Using Polystyrene Spheres Spin-Coating Method for PERC Solar Cells

**DOI:** 10.3390/ma9070549

**Published:** 2016-07-08

**Authors:** Chia-Hsun Hsu, Chih-Hsiang Yang, Yi-Han Wang, Chun-Wei Huang, Shui-Yang Lien, Chung-Yuan Kung, Jen-Chung Lou

**Affiliations:** 1 Department of Materials Science and Engineering, Da-Yeh University, Chunghua 51591, Taiwan; cstcaptive@gmail.com; 2 Department of Electrical Engineering, National Chung Hsing University, Taichung 40227, Taiwan; calvin.y630@gmail.com (C.-H.Y.); cykung@nchu.edu.tw (C.-Y.K.); 3 Electronics and Communication Engineering, Peking University, Wuxi 214125, China; snoopy25402@gmail.com (Y.-H.W.); Jesselou@ss.pku.edu.cn (J.-C.L.); 4 Department of Electrical Engineering, Da-Yeh University, Chunghua 51591, Taiwan; oscarhuang1992@gmail.com

**Keywords:** passivated emitter and rear cell, local contact opening, polystyrene sphere

## Abstract

The passivated emitter and rear cell (PERC) concept is one of the most promising technologies for increasing crystalline silicon solar cell efficiency. Instead of using the traditional laser ablation process, this paper demonstrates spin-coated polystyrene spheres (PS) to create local openings on the rear side of PERCs. Effects of PS concentration and post-annealing temperature on PERC performance are investigated. The experimental results show that the PS are randomly distributed on wafers and no PS are joined together at a spin rate of 2000 rpm. The PS can be removed at a temperature of 350 °C, leaving holes on the passivation layers without damaging the wafer surfaces. As compared to the laser opening technique with the same contact fraction, the PS opening technique can yield a higher minority effective lifetime, a higher implied open-circuit voltage, and a slightly higher short-circuit current. Although the fill factor of the PS opening technique is lower owing to non-optimized distribution of the openings, the conversion efficiency of the devices is comparable to that of devices prepared via the laser opening process.

## 1. Introduction

Passivated emitter and rear cells (PERCs) are considered a next-generation monocrystalline silicon (c-Si) solar technology owing to their significant efficiency gain without a large increase in the manufacturing costs on traditional industrial production lines [1,2]. The most obvious difference between typical c-Si solar cells and PERCs is the rear side passivation layers, which not only efficiently reduce the recombination velocity, but also reflect longer wavelengths on the rear side surfaces [3,4,5]. To create a rear side metal contact, laser ablation technology is used to remove passivation layers to produce local openings [6,7]. Laser ablation might cause unintended laser-induced damage, such as silicon recrystallization, surface melting, and heat-affected zones that diminish cell performance [8]. To achieve damage-free openings, shorter laser pulse widths in the range of picoseconds to femtoseconds are used [1]. The picosecond or femtosecond lasers are around seven to ten times more expensive than nanosecond laser systems. Development of a cheaper method to replace conventional laser technology is necessary.

In this work, we demonstrate an opening technique using polystyrene spheres (PS). The morphologies of different concentrations of PS spin-coated on wafers are shown. The effect of temperature on the PS removal process is investigated. Finally, the performance of PERCs prepared using the PS opening technique is presented.

## 2. Experimental Methods

**Figure 1:**
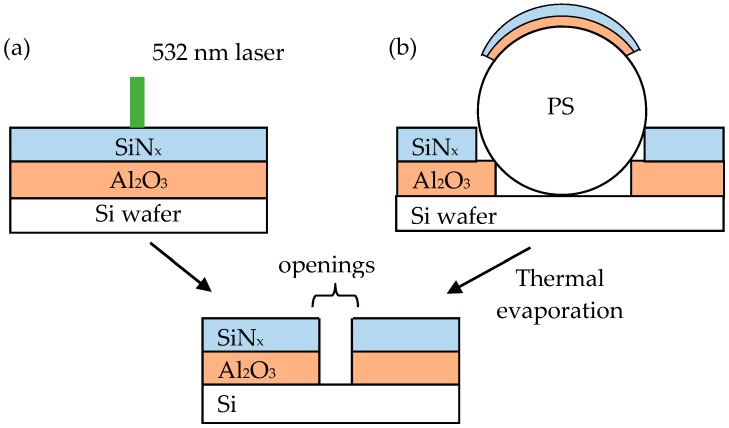
Diagram of (**a**) laser and (**b**) polystyrene spheres (PS) opening technique for passivated emitter and rear cells (PERCs).

The solution contained 0.5 wt. % PS in deionized water were obtained from Polysciences, Inc. (Warrington, PA, USA) The diameter of the PS was 40 µm. The solution was diluted to the concentrations to 0.15–0.3 wt. % by adding ethanol. No surfactant was used. The p-type silicon wafer had a doping concentration of 10^16^ cm^−3^, size of 15.6 × 15.6 cm^2^, thickness of 200 µm, and resistivity of 1 Ω-cm. The wafers were cleaned by standard Radio Corporation of America, process consisting of immersion steps in standard cleaning 1 (5:1:1 H_2_O:NH_4_OH:H_2_O_2_), 1% hydrofluoric acid, and standard cleaning 2 (6:1:1 H_2_O:HCl:H_2_O_2_) solutions to remove organic contamination, particles and native silicon oxide on the surface. Some wafers were sliced into small pieces of 2 × 2 cm^2^. Then, the PS opening technique was performed as shown in Figure 1. The PS solutions with concentrations ranging from 0.15 to 0.3 wt. % were applied on the wafer surface manually and statically prior to spin coating. The spin velocity was initially 500 rpm for 30 s and increased up to 2000 rpm for 60 s. The effect of the PS concentration on the morphological distribution was investigated. A 10-nm aluminum oxide (Al_2_O_3_) layer and a 150-nm silicon nitride (SiN_x_) layer were deposited by atomic layer deposition at room temperature and by plasma-enhanced chemical vapor deposition at 120 °C on wafers, as well as on PS. Afterwards, the samples were loaded into a thermal furnace at atmospheric pressure with temperatures of between 150 °C and 450 °C for 30 min in order to evaporate the PS from the wafers. Part of the overlying Al_2_O_3_ was lifted off leaving local holes.

PERCs with a typical front-side structure of Ag/SiN_x_/SiO_2_/n emitter/p wafer base were fabricated. The PS opening technique was applied to the rear side of the devices to create local holes. The emitter sheet resistance was 75 Ω/sq. An Al layer with a thickness of 200 nm was sputtered on the rear side of the device and this contacted with silicon wafer through the holes. The devices were annealed in a furnace with a peak temperature of 800 °C, which was held for about 15 s, to form a back surface field. The morphologies of the wafers were observed by optical microscopy (OM) (M&T Optics, Co., Ltd., Taipei, Taiwan). The minority carrier lifetime and implied open-circuit voltage (implied V_oc_) of the wafers were determined by quasi-steady-state photoconductance (WCT-120, Sinton Consulting Inc., Boulder, CO, USA) at 25 °C. The J–V characteristics of the PERCs were measured using a standard solar simulator under AM1.5G (100 mW/cm^2^) conditions.

## 3. Results and Discussion

**Figure 2:**
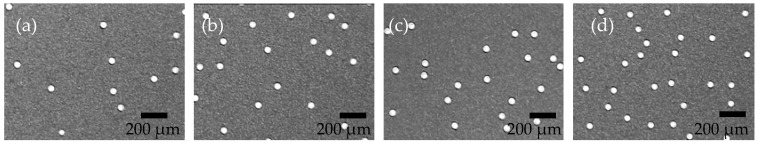
Optical microscopy (OM) images for the wafers with PS concentrations of (**a**) 0.15; (**b**) 0.2; (**c**) 0.25 and (**d**) 0.3 wt. % at a spin speed of 2000 rpm.

Figure 2 shows the OM topographical images of the wafers spin-coated with PS concentrations of (a) 0.15; (b) 0.2; (c) 0.25; and (d) 0.3 wt. %. The number of PS per unit area increases with increasing PS concentration. It can be clearly seen that the PS are separated in all the cases. This is attributed to the two-stage spin-coating process used. Empirically, the extent of the separation of the PS is positively related to the spin speed of the second stage. In the present study, the maximum spin speed used was 2000 rpm owing to equipment limitations, but it resulted in the isolation of every PS. The coverage ratio of PS area to wafer area was calculated by using Image Pro computer software. The coverage ratios for the PS concentrations of 0.15, 0.2, 0.25, and 0.3 wt. % were 1.51%, 2.45%, 2.88% and 3.98%, respectively. These coverage ratios are also in the range of those obtained from typical PERCs prepared with the laser opening process.

**Figure 3:**
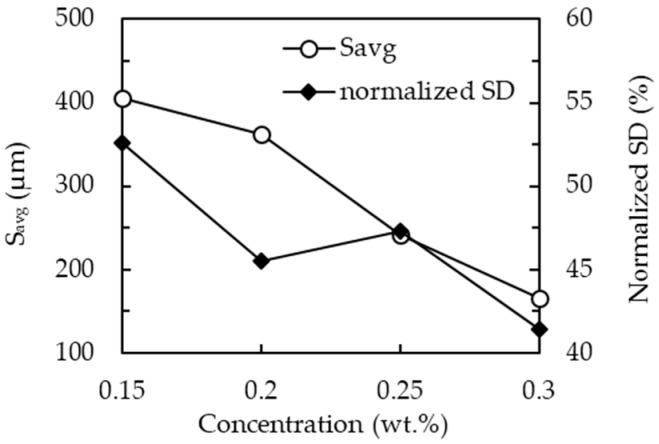
Average PS spacing, S_avg_, and normalized SD for the different PS concentrations.

The average spacing, S_avg_, of the PS is calculated by the following equation:

(1) $$S_{\text{avg}}=\frac{1}{n}\overset{n}{\underset{i=1}{\sum }}\frac{\left(d_{i1}+d_{i2}+d_{i3}+d_{i4}\right)}{4}$$

where n is the total number of PS and d_i1_, d_i2_, d_i3_, and d_i4_ are the distances between a PS and its four nearest neighbors. The calculation results are shown in Figure 3. The value of S_avg_ reduces from 410 to 163 µm when the PS concentration increases from 0.15 to 0.3 wt. %. The normalized standard deviation (SD), defined as SD divided by S_avg_, can be determined, as shown in Figure 2, to help evaluate the uniformity of PS distribution. The normalized SDs are 52.6%, 45.5%, 47.3%, and 41.4% for PS concentrations of 0.15, 0.2, 0.25, and 0.3 wt. %, respectively. The large coefficient of variation in PS particle distribution might possibly be further narrowed by adding surfactant to the PS solution, or by optimizing parameters of the spin coating process. The non-uniformly distributed openings may have a significant influence if the surface recombination is high. However, the aluminum oxide (Al_2_O_3_) layer deposited by using atomic layer deposition (ALD), which has been reported as the greatest passivation layer currently available, can yield a very low surface recombination velocity. Adverse influences related to the PS non-uniformity can thus be significantly reduced.

**Figure 4:**
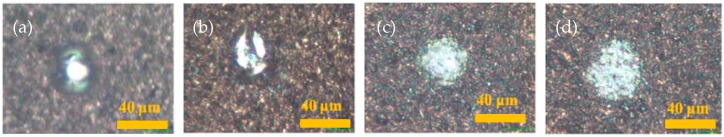
OM images for the PS with annealing temperatures of (**a**) 150 °C; (**b**) 250 °C; (**c**) 350 °C and (**d**) 450 °C.

A 10-nm-thick Al_2_O_3_ passivation layer and a 150-nm-thick SiN_x_ capping layer were deposited on the wafer as well as PS, followed by an annealing process with temperatures ranging from 150 °C to 450 °C for 30 min. Figure 4 shows the OM images for the annealing processes of (a) 150 °C; (b) 250 °C; (c) 300 °C; and (d) 350 °C. It can be seen that at the temperature of 150 °C no significant change could be observed between the OM images before and after annealing. The PS remain on the wafer surface. At 250 °C, the PS are found to partly decompose. When the temperature is 350 °C and 450 °C, no PS are observable on the wafers. Temperatures above 350 °C can provide sufficient energy to not only evaporate the PS but also push out the overlying films. No damage to the wafers can be reasonably expected and assumed. In contrast, in the case of the traditional laser opening process, the Al_2_O_3_ and SiN_x_ layers have very small absorption of laser energy, so the strategy for creating holes is that the Si wafer absorbs heat energy, which would then be transferred by conduction in a few nanoseconds to the adjacent layers [9,10,11]. The ablation of the passivation layer initiates when the temperature reached in the layers is of the order of the boiling temperature (around 3525 K for Al_2_O_3_ and 2150 K for SiN_x_) [12]. The required laser energy is about 0.8 J/cm^2^, which is close to the energy (typically around 1 J/cm^2^) that can melt or damage Si wafers [12]. It is noted that there might be some kind of carbon trace remaining on the surface wafer, and this would possibly affect metal contact formation. The Al contact was sputtered on the wafers, and the specific contact resistance (ρ_c_) between Al and Si wafer was determined by using transmission line method. ρ_c_ of the sample annealed at 450 °C is 8.4 × 10^−3^ Ω-cm^2^, which is comparable to the value (8.6 × 10^−3^ Ω-cm^2^) of the sample prepared with the laser method. As a result, the effect of the residue of carbon trace at the wafer surface might be insignificant.

**Figure 5:**
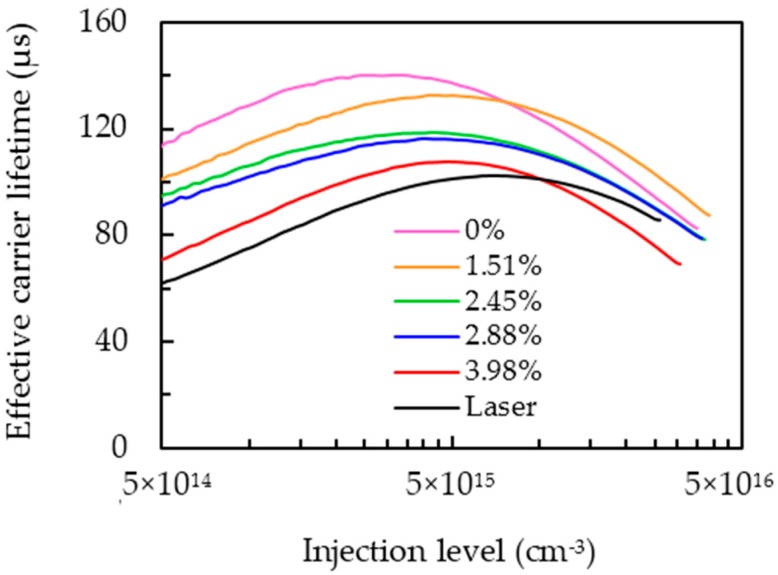
Effective carrier lifetime as a function of injection level.

**Figure 6:**
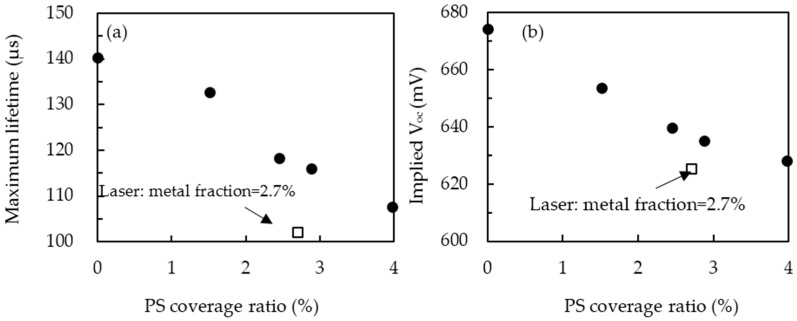
(**a**) Maximum effective carrier lifetime and (**b**) implied V_oc_ values as a function of PS coverage ratio. Wafers with identical structure but using laser opening technique is indicated.

Figure 5 shows the effective carrier lifetime as a function of injection level for the wafers prepared with different PS coverage ratios after PS annealing process. The maximum carrier lifetime for each curve is illustrated in Figure 6a. With no PS on the wafer, corresponding to the coverage ratio of 0 wt. %, the highest lifetime of 140 µs can be reached owing to the full surface passivation by Al_2_O_3_. The lifetime reduces from 132 to 107 µs as the coverage ratio is increased from 1.51% to 4.57%. The reduction can be directly attributed to the loss in passivation area caused by an increase in the amount of local holes when the coverage ratio increases. For the purpose of comparison, the wafer with the laser openings was fabricated with a hole diameter of 40 µm and spacing of about 240 µm, corresponding to a metal fraction of 2.7%. The lifetime of the wafer with the laser openings is 103 µs, lower than that of the PS technique if the same metal fraction is used.

The implied V_oc_ for the wafers prepared with the PS opening technique is shown in Figure 6b to evaluate the maximum V_oc_ before metal deposition. The implied V_oc_ is determined as given by [13]:

(2) $$\tau_{\text{eff}}\left(∆n\right)=\frac{∆n\left(t\right)}{G-∆n\left(t\right)/∆t}$$

(3) $${\text{implied V}}_{\text{oc}}=\frac{\text{kT}}{q}\ln\left(\frac{∆n\left(N_{A}+∆n\right)}{{n_{i}}^{2}}\right)$$

where G is the generation rate; τ_eff_ is the effective minority carrier lifetime; ∆n is the excess carrier concentration; ∆t is the time taken in seconds; k is the Boltzmann constant; T is the temperature; N_A_ is the acceptor concentration of the wafer; and n_i_ is the intrinsic carrier concentration. The implied V_oc_ follows the same trend as the effective minority carrier lifetime with respect to the PS concentration. It should be noted that the wafer prepared with the laser opening technique has an implied V_oc_ of 619 mV, whereas the wafer prepared with the PS opening technique should be around 628–633 mV if it had the same metal fraction.

**Figure 7:**
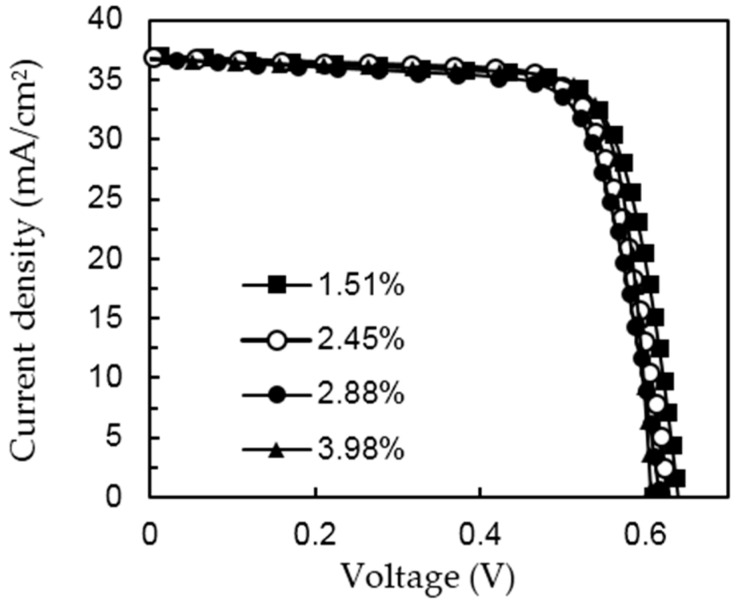
J–V characteristics of the PERCs for the different PS coverage ratio.

Figure 7 shows the J–V characteristics of the PERCs with different PS coverage ratios. The detailed solar cell external parameters are summarized in Table 1. It can be seen that the conversion efficiency trade-off is the gain in FF and the loss in V_oc_ when the PS coverage ratio increases. FF can be related to the series resistance (R_s_) and the shunt resistance (R_sh_) of the devices, which could be calculated from the inverse slopes of the J–V curves at V = 0 and J = 0, respectively. The result shows that most of the gain in FF is attributed to the reduced R_s_, resulting from the increased contact area between the silicon wafer and the back electrode as the coverage ratio increases. FF for the PS method with a metal fraction of 2.88% is higher than that for the laser method with the coverage ratio of 2.7%, might be attributed to the non-uniform PS distribution. The reduction in V_oc_ is owing to the loss in passivation area, which is supported by the lifetime measurement. J_sc_ slightly decreases with the PS coverage ratio, presumably owing to the carrier loss at the rear surface. Overall, the optimal conversion efficiency of 17.94% occurs at the coverage ratio of 2.88%. Twenty devices were reproduced under the same conditions, and the conversion efficiencies were between 17.8% and 17.95% with an average of 17.92%. This result demonstrates that the PS opening technique could yield cell efficiencies comparable to that of the PERC cell with identical structure but prepared with the laser opening technique, which is 17.99% (V_oc_ = 605 mV, J_sc_ = 36.9 mA/cm^2^, FF = 80.6%).

## 4. Conclusions

This study uses PS to create local openings for rear contacts of PERCs. The PS are randomly distributed on wafers, and the PS are isolated at a spin rate of 2000 rpm. Annealing temperatures of greater than 350 °C for 30 min can completely remove the PS, leaving holes on the passivation layer. The PS annealing process can thus be combined with the annealing process for the SiN_x_/Al_2_O_3_ passivation layer, so no additional annealing process is needed. The best PERC cell efficiency obtained with the PS opening technique is 17.94%, which comparable to that of the cell with identical structure prepared using the laser opening technique.

## Figures and Tables

**Table 1:** Performance of the PERCs with different PS coverage ratios.

Coverage (%)	V_oc_ (mV)	J_sc_ (mA/cm^2^)	FF (%)	η (%)	R_s_ (Ω-cm^2^)	R_sh_ (Ω-cm^2^)
1.51	641	37.2	74.7	17.81	2.03	435.6
2.45	629	37	76.7	17.85	1.88	422.1
2.88	622	36.9	78.2	17.94	1.51	420.7
3.98	609	36.6	79.4	17.69	0.93	410.2
2.7 (laser)	605	36.9	80.6	17.99	1.36	399.7
